# Tracheo-brachiocephalic artery fistula after tracheostomy associated with thoracic deformity: a case report

**DOI:** 10.1186/1752-1947-5-595

**Published:** 2011-12-28

**Authors:** Kie Ogawa, Norihisa Nitta, Akinaga Sonoda, Masashi Takahashi, Tomoaki Suzuki, Shoji Kitamura, Jun Hanaoka, Noriaki Tezuka, Kiyoshi Murata

**Affiliations:** 1 Department of Radiology, Shiga University of Medical Science, Tsukinowa-cho Seta Otsu Shiga, 520-2192, Japan; 2 Department of Surgery, Shiga University of Medical Science, Tsukinowa-cho Seta Otsu Shiga, 520-2192, Japan

## Abstract

**Introduction:**

Tracheo-brachiocephalic artery fistulae are critical long-term complications after tracheostomy, reported in 0.6% of patients within three to four weeks after the procedure. In 30% to 50% of cases there is some bleeding prior to onset. Since the onset involves sudden massive bleeding, the prognosis is poor; the reported survival rate is 10% to 30%. The direct cause of bleeding is the formation of a fistula with the trachea subsequent to arterial injury by the tracheostomy tube. Endo-tracheal factors are movement of the tracheostomy tube due to body movement and seizures, pressure exerted by the cuff of the tracheostomy tube, tracheostomy at lower levels, and the fragility of blood vessels and the trachea due to steroid or radiation therapy, and malnutrition. Extra-tracheal factors include prior surgery and deformity and shifting of the trachea and major blood vessels due to congenital kyphoscoliosis or thoracic deformity. There has been no report of the usefulness of contrast-enhanced computed tomography studies to identify the anatomical relationship between the trachea and brachiocephalic artery.

**Case presentation:**

A 27-year-old Mongolian woman with congenital muscular dystrophy who underwent tracheal intubation for airway management due to pneumonia and granulation development developed a tracheo-brachiocephalic artery fistula during the placement of the tracheostomy tube. It was diagnosed by contrast-enhanced chest computed tomography and repaired. About a month later she developed massive airway bleeding during replacement of the tracheostomy tube. Temporary hemostasis was achieved by compression via cuff inflation. A contrast-enhanced chest computed tomography scan demonstrated a narrowed brachiocephalic artery running along and ventral to the tube and a tracheo-brachiocephalic artery fistula was suspected. She underwent brachiocephalic artery resection and aorta, right common carotid artery, and subclavian artery bypass surgery with an innominate vein, tracheoplasty, and partial sternectomy. We noted marked thoracic deformity; the brachiocephalic artery was compressed by the trachea and chest wall resulting in localized wall necrosis and the development of a tracheo-brachiocephalic artery fistula, a fatal complication whose prevention is important.

**Conclusions:**

We suggest that before tracheostomy, the anatomic relationship between the trachea and brachiocephalic artery must be confirmed by contrast-enhanced chest computed tomography scan.

## Introduction

Tracheo-brachiocephalic artery fistulae are a rare but critical, life-threatening complication of tracheostomy that must be prevented. Causal factors vary; in patients with thoracic deformity, computed tomography (CT) studies can help to identify the anatomical relationship between the trachea and brachiocephalic artery. We report the case of a patient whose tracheo-brachiocephalic artery fistula was diagnosed by contrast-enhanced chest CT and whose life was saved.

## Case presentation

Our patient was a 27-year-old Mongolian woman with mental retardation since birth. She developed pneumonia once or twice a year and was repeatedly admitted to hospital. She had experienced progressive weakening of muscle strength since 2009 and genetic testing in our pediatrics department returned a diagnosis of congenital myotonic dystrophy. A year later, she again developed pneumonia and was admitted to a nearby hospital where she underwent tracheal intubation for respiratory management. Her pneumonia responded to treatment with antibiotics, however, ventilation insufficiency was observed during tracheal intubation and she was transferred to another hospital after one month. She experienced respiratory arrest despite respirator management. Bronchoscopy confirmed obstruction of the tracheal tube by granuloma. Endo-tracheal inspection revealed stenosis and deformity of the trachea and airway maintenance was difficult. Consequently, she was transferred to our Respiratory Surgery Department for endo-tracheal stenting after two weeks.

She had undergone laryngeal and middle ear surgery at the age of 10; further details of this were unknown. She was diagnosed as having ileus and congenital myotonic dystrophy at the age of 25.

**Figure 1:**
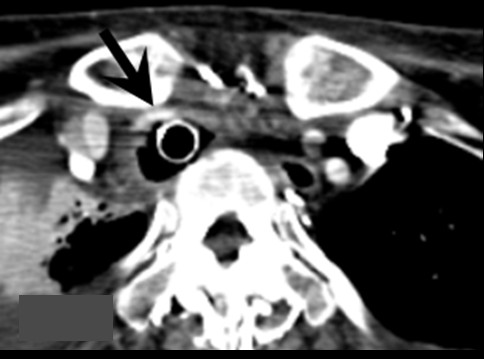
**The narrowed brachiocephalic artery runs along and ventral to the tube (arrow)**. Although computed tomography detected no evidence of active bleeding, a tracheo-brachiocephalic artery fistula was suspected due to arterial bleeding.

As we expected that she would have difficulty in coughing up sputum we postponed endo-tracheal stenting. One day after transferring to our Respiratory Surgery Department, she underwent tracheostomy and a long tracheal tube was inserted to maintain the airway. She subsequently developed anemia that responded to iron supplementation. One month later, we performed percutaneous endoscopic gastroscopy; this improved her nutritional management. Five days after that, during replacement of the tracheostomy tube, she developed massive arterial bleeding; hemostasis was achieved by compression with the inflated cuff. Contrast-enhanced chest CT findings (Figure 1) led us to suspect a tracheo-brachiocephalic artery fistula and she underwent surgery at our Cardiovascular Surgery Department.

**Figure 2:**
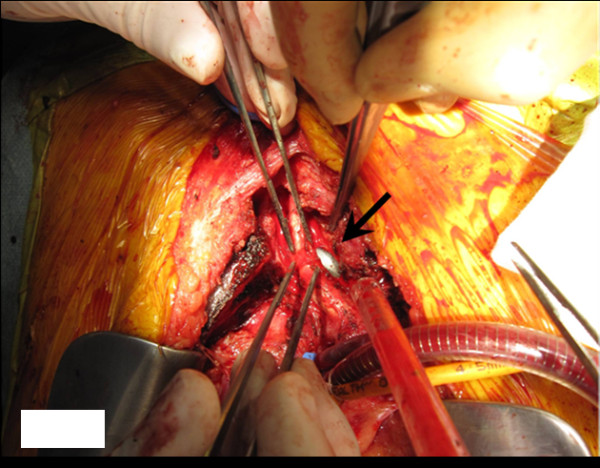
**Intra-operative photograph**. The cuff of the intubation tube is seen at the site where the brachiocephalic artery branches off the trachea (arrow). Note the tear in the brachiocephalic artery.

Intra-operatively, the cuff of the tracheostomy tube was exposed at the site where the right brachiocephalic artery adhered to the trachea. When we lifted the vessel carefully we found a 1.5-cm-long tear (Figure 2) whose direct closure was difficult because the adjacent wall was fragile. We also thought it necessary to obtain some distance from the trachea. We performed brachiocephalic artery resection and aorta, right common carotid artery, and subclavian artery bypass surgery with an innominate vein, tracheoplasty, and partial sternectomy. The bypassed area was protected with pericardium and adjacent tissue.

**Figure 3:**
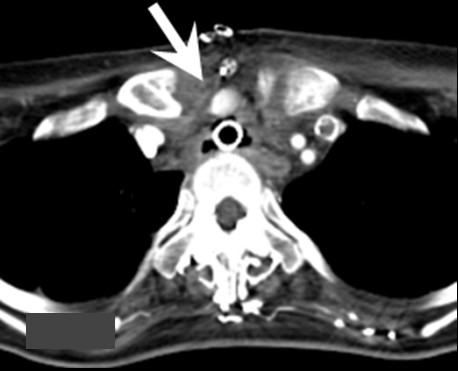
**The brachiocephalic artery running along the upper part of the sternum**. Resection of this part of sternum rendered the space between the trachea and brachiocephalic artery wider and increased the diameter of the brachiocephalic artery (arrow).

A post-operative contrast-enhanced chest CT scan revealed that the space between the trachea and brachiocephalic artery was wider and the diameter of the brachiocephalic artery was larger than before the procedures (Figure 3). At 12 days after surgery, she was discharged from intensive care unit (ICU) because her general condition had improved. She continued to receive respiratory assistance via peroral tracheal intubation; when the tracheal tube was shifted, she sometimes developed ventilation insufficiency probably due to tracheal stenosis. Then, 10 days after she was discharged from the ICU, she again underwent tracheostomy that elicited no events.

Since there was a stenotic area just above the tracheal bifurcation we chose to place a long tracheostomy tube; this rendered airway maintenance possible. Five days after placement of the tracheostomy tube, a contrast-enhanced chest CT scan revealed obstruction at the brachiocephalic artery bypass. There were no obvious neurological findings. To avoid endo-tracheal bleeding we did not administer anti-coagulation therapy.

A granuloma had developed at the tip of the tracheostomy tube just above the tracheal bifurcation, and she occasionally experienced episodes of choking. Deep insertion of the tracheostomy tube produced one-lung ventilation. She underwent tracheal stenting under general anesthesia to alleviate the airway obstruction due to granuloma formation after two weeks. A shorter tracheostomy tube was inserted and placed within the stent. She was subsequently able to breathe on her own without a respirator; her tracheostomy tube could be replaced smoothly. Continued rehabilitation improved her general condition and she was discharged and transferred to a hospital close to her home three months after admission.

## Discussion

We report a patient with a thoracic deformity who had developed a tracheo-brachiocephalic artery fistula. Our patient had massive bleeding from the tracheal wall when tracheostomy tubes were exchanged. The bleeding was stopped by re-inflation of the tracheostomy cuff, after which an emergency contrast-enhanced CT scan revealed a tracheo-brachiocephalic artery fistula that was managed by surgical vascular reconstruction. Based on the CT scan finding that the brachiocephalic artery was markedly narrow and was compressed by both the intra-tracheal cuff and the clavicle, we concluded that the tracheo-brachiocephalic artery fistula was caused by cuff-induced pressure necrosis. It is critical in the prevention of a potentially fatal complication to appreciate that in patients with a thoracic deformity who have undergone tracheostomy a fistula might form between the brachiocephalic artery and trachea. Confirmation by contrast-enhanced CT scan of the positional relationship between the intra-tracheal cuff and the brachiocephalic artery as well as the degree of compression or displacement of the artery are extremely important.

**Figure 4:**
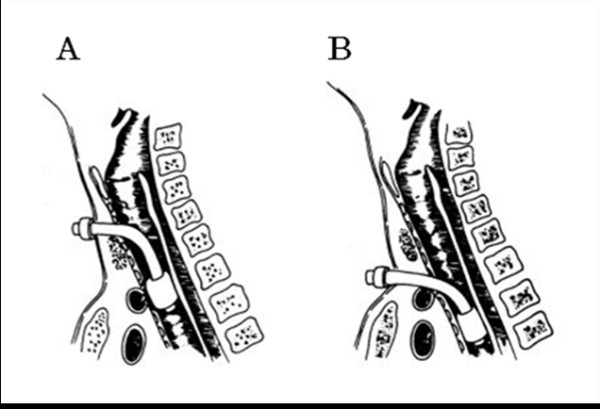
**Types of trachea-brachiocephalic artery fistula**. **(A) **Extra-tracheal type and **(B) **endo-tracheal type (modified from Jones *et al. *[1]).

Tracheo-brachiocephalic artery fistulae are critical long-term complications after tracheostomy and have been reported in 0.6% of patients [1] within three to four weeks after the procedure [2]. In 30% to 50% of cases there is some bleeding prior to onset [1]. Since the onset is sudden massive bleeding, the prognosis is poor; the reported survival rate is 10% to 30% [3]. The direct cause of bleeding is the formation of a fistula with the trachea subsequent to arterial injury by the tracheostomy tube. Endo-tracheal factors are movement of the tracheostomy tube due to body movement and seizures [2,4], pressure exerted by the cuff of the tracheostomy tube [5], tracheostomy at lower levels [6], and the fragility of blood vessels and the trachea due to steroid or radiation therapy, and malnutrition [7]. Extra-tracheal factors include prior surgery and deformity and shifting of the trachea and major blood vessels due to congenital kyphoscoliosis or thoracic deformity [8]. Based on the onset mechanism, bleeding from the brachiocephalic artery after tracheostomy is categorized as extra-tracheal and endo-tracheal (Figure 4) [1]. In patients with extra-tracheal type the tracheostomy tube compresses and injures the brachiocephalic artery, resulting in bleeding at the lower anterior part of the tracheostoma. The endo-tracheal type is due to compression and necrosis of the trachea induced by the tip of the tracheal tube and cuff which creates a tracheo-brachiocephalic artery fistula and leads to bleeding.

To save the patient's life in these circumstances, urgent temporary hemostasis must be achieved by over-inflation of the cuff of the tracheostomy tube [1], direct compression by fingers inserted through the tracheostoma, or peroral intubation and replacement of the tracheostomy tube with a peroral tracheal tube in combination with compression of the sternum from above [9]. Percutaneous endovascular stent-graft placement and embolization have been reported in the management of patients with trachea-brachiocephalic artery fistulae. While hemostasis can be achieved by surgical techniques, for example, suturing the fistula closed and transplanting artificial blood vessels and alternative veins, approximately 10% of patients have neurological sequelae. Recently, Iodice *et al. *reported that they successfully performed preventive ligation of the brachiocephalic artery for seven cases with neuromuscular disorders at risk for trachea-brachiocephalic artery fistula and pre-operative contrast-enhanced CT provided useful information on the structural relationship between the trachea and the brachiocephalic artery [10]. As the focus should be on prevention rather than treatment, the presence or absence of thoracic deformity and of tracheal stenosis must be identified and the anatomical relationship between the trachea and cardiovascular system must be elucidated by contrast-enhanced CT studies prior to undertaking tracheostomy procedures.

## Conclusions

Tracheo-brachiocephalic artery fistulae are a fatal complication of tracheostomy and must be prevented. In patients with thoracic deformity it is necessary to confirm the anatomical relationship between the trachea and brachiocephalic artery by contrast-enhanced chest CT before undertaking tracheostomy.

## Consent

Written informed consent was obtained from the patient's next-of-kin for publication of this case report and any accompanying images. A copy of the written consent is available for review by the Editor-in-Chief of this journal.

## Competing interests

The authors declare that they have no competing interests.

## Authors' contributions

OK and AS interpreted chest X-ray and CT images and made the diagnoses. NN was instrumental in the preparation of the manuscript. TS, SK, JH and NT performed surgical repair and tracheal stenting for our patient. SK and JH obtained informed consent from our patient's next-of-kin. NN, MT and KM edited this article. All authors read and approved the final manuscript.
